# Cancer vaccines: advances, hurdles, and future directions

**DOI:** 10.37349/etat.2025.1002350

**Published:** 2025-11-24

**Authors:** Panagiotis J. Vlachostergios

**Affiliations:** University of Salford, UK; ^1^Department of Medical Oncology, IASO Thessalias Hospital, 41500 Larissa, Greece; ^2^Division of Hematology and Medical Oncology, Weill Cornell Medicine, New York, NY 10065, USA

**Keywords:** cancer vaccines, neoantigen, mRNA vaccine, autogene cevumeran, personalized immunotherapy, neoepitope discovery

## Abstract

Therapeutic cancer vaccines harness the adaptive immune system to eradicate malignancies by targeting tumor-specific antigens. This review charts the evolution of cancer vaccine platforms—from shared tumor-associated antigens (TAAs) and dendritic cell (DC) vaccines to next-generation neoantigen-messenger ribonucleic acid (mRNA) vaccines—highlighting advances in vaccine delivery, antigen discovery, computational prediction, and translational efficacy. We explore cutting-edge clinical data, including long-lived T-cell memory and promising outcomes in various cancer types, including pancreatic ductal adenocarcinoma (PDAC), melanoma, head and neck cancers, renal cell carcinoma (RCC), and others. We address critical challenges, including tumor heterogeneity, manufacturing scalability, biomarker development, and regulatory frameworks, and propose an integrated translational ecosystem to accelerate the adoption of personalized cancer vaccines.

## Introduction

Therapeutic cancer vaccines represent a rapidly advancing form of precision immunotherapy that differs fundamentally from prophylactic vaccines, which are designed to prevent infectious diseases or virus-associated cancers before they occur. In the therapeutic context, the goal is to educate and direct the host’s immune system—particularly cytotoxic CD8⁺ T lymphocytes and helper CD4⁺ T cells—to specifically recognize tumor-derived antigens and eradicate malignant cells, including micrometastatic disease. Early generations of cancer vaccines, which primarily targeted tumor-associated antigens (TAAs) such as MUC1, HER2, or prostate acid phosphatase (PAP), often failed to achieve durable clinical benefit due to immune tolerance mechanisms, antigen heterogeneity, and the low intrinsic immunogenicity of self-derived proteins. Moreover, delivery systems of the time struggled to achieve efficient antigen presentation and robust T-cell priming.

Over the past decade, however, a convergence of technological innovations—including high-throughput next-generation sequencing, advanced immunopeptidomics, predictive bioinformatics pipelines, and flexible delivery platforms such as lipid nanoparticle (LNP)-encapsulated messenger ribonucleic acid (mRNA)—has fundamentally transformed the field. These tools enable the rapid identification of patient-specific tumor neoantigens arising from somatic mutations, the design of individualized vaccine constructs, and the induction of potent, polyclonal, and long-lived T-cell responses. As a result, cancer vaccines are transitioning from a niche experimental therapy to a clinically viable pillar of personalized oncology, with multiple trials now demonstrating promising efficacy in traditionally hard-to-treat malignancies [1, 2].

## Platforms and antigen prioritization

### TAAs vs. neoantigens

TAAs are proteins or glycoproteins that are expressed at higher levels in tumor tissue compared to normal tissues, or are aberrantly expressed during oncogenesis [3]. Classic examples include cancer-testis antigens such as MAGE-A family members, and tissue-specific proteins like PAP in prostate cancer. Because TAAs are often shared among patients with the same cancer type, they lend themselves to “off-the-shelf” vaccine approaches that can be manufactured at scale and distributed broadly without the need for patient-specific customization [3]. However, their origin from normal self-proteins means that they are subject to central and peripheral tolerance mechanisms, leading to limited T-cell repertoire diversity and relatively weak immunogenicity. In contrast, neoantigens arise from non-synonymous somatic mutations in tumor DNA that create novel peptide sequences absent from the normal human proteome [3]. These alterations, which can result from point mutations, insertions/deletions, or gene fusions, are processed and presented by major histocompatibility complex (MHC) molecules on tumor cells, where they are recognized as “non-self” by the immune system [3]. Because neoantigens bypass central tolerance, they have the potential to elicit high-avidity T-cell responses capable of selective tumor destruction without significant off-target toxicity. The patient-specific nature of most neoantigens makes them ideal for truly personalized vaccine design, where next-generation sequencing and computational epitope prediction are used to identify and prioritize targets most likely to generate effective antitumor immunity [3, 4]. Table 1 summarizes TAAs, neoantigens, and cryptic antigens (a newer category that includes products of gene fusions or non-canonical translation events), along with the pros and cons of vaccinating against each antigen.

**Table 1:** Comparison of tumor antigen classes and their vaccination pros/cons.

**Antigen type**	**Examples**	**Definition**	**Pros of vaccination**	**Cons of vaccination**
Tumor-associated antigens (TAAs)	MUC1, HER2, prostate acid phosphatase (PAP), MAGE-A, NY-ESO-1	Self-antigens overexpressed or aberrantly expressed in tumors but are also found at low levels in normal tissue	- Broadly shared across patients - Suitable for “off-the-shelf” vaccines - Scalable manufacturing	- Subject to immune tolerance - Weak immunogenicity - Risk of autoimmunity if expressed in normal tissues
Neoantigens	KRAS G12D, TP53 R175H, BRAF V600E, PIK3CA E545K, EWS-FLI1, PAX7-FOXO1 fusions	Non-self antigens arising from somatic mutations or gene fusions unique to tumor cells	- High immunogenicity - No central tolerance - Personalized and tumor-specific - Lower off-target risk	- Individualized production (time-consuming and costly) - Requires tumor sequencing and prediction - May miss escape variants
Cryptic/Hidden antigens (e.g., fusion breakpoints)	EWSR1 exon 7-FLI1 fusion (e.g., “SQQSSSYGQQ...”)	Peptides derived from untranslated regions, aberrant transcripts, or fusion breakpoints absent from the normal proteome	- Highly specific to tumors - Potential “public” neoantigens in some sarcomas - May be shared across subsets of patients	- Limited validation - Often unknown expression/presentation - Technical difficulty in identification and prediction

### Vaccine delivery modalities

A wide spectrum of delivery platforms has been explored for cancer vaccines, each with distinct advantages and limitations that influence their clinical applicability. Peptide-based vaccines are conceptually straightforward: synthetic short or long peptides representing tumor antigens are administered with immune-stimulating adjuvants to elicit T-cell responses [5]. While they allow precise control over antigen composition and can be manufactured relatively easily, peptides alone are typically weakly immunogenic and require potent adjuvants—such as toll-like receptor (TLR) agonists, Montanide emulsions, or poly-ICLC—to effectively prime CD8⁺ and CD4⁺ T-cell responses. DNA vaccines, consisting of plasmids encoding tumor antigens, are stable, inexpensive to produce, and well-suited to large-scale distribution. However, in human trials they have generally induced limited immunogenicity without additional delivery enhancements (e.g., electroporation or molecular adjuvants).

mRNA vaccines, particularly those encapsulated in LNPs, have emerged as a leading platform in recent years due to their ability to induce robust antigen expression in host cells and present both class I and class II epitopes, thereby activating cytotoxic CD8⁺ and helper CD4⁺ T cells simultaneously [5]. The modular nature of mRNA manufacturing enables rapid adaptation to individual patients’ neoantigen repertoires, and lessons learned from the COVID-19 mRNA vaccine rollout have accelerated scalability and regulatory familiarity [5, 6].

Dendritic cell (DC)-based vaccines involve isolating autologous DCs from the patient, loading them ex vivo with tumor-associated or neoantigen peptides, and then re-infusing them to initiate potent T-cell priming [7, 8]. This platform directly harnesses the body’s most effective antigen-presenting cells (APCs), and early melanoma studies demonstrated the capacity of DC vaccines to generate durable T-cell memory and sustained clinical responses [7, 8].

Oncolytic virus vaccines add a cytolytic dimension to immunotherapy by infecting and lysing tumor cells while delivering tumor antigens in an inflammatory context. The leading example, talimogene laherparepvec (T-VEC), is an engineered herpes simplex virus type 1 (HSV-1) expressing granulocyte-macrophage colony-stimulating factor (GM-CSF). FDA-approved for advanced melanoma, T-VEC not only mediates direct tumor cell lysis but also promotes systemic antitumor immunity and has shown synergistic effects when combined with immune checkpoint inhibitors [9–11].

Heat shock protein (HSP)-based vaccines exploit the natural chaperoning of tumor-derived peptides by HSPs to APCs, thereby enhancing cross-presentation and T-cell activation [12]. Because the peptide cargo is derived directly from the patient’s tumor, this approach offers an inherently personalized antigen repertoire. However, logistical challenges—including the need for tumor tissue procurement and complex purification processes—have limited their broader clinical adoption despite promising immunogenicity signals in early-phase studies [12].

## Translational advances and clinical evidence

### Personalized mRNA vaccines: the paradigm of autogene cevumeran for pancreatic cancer

Autogene cevumeran (a personalized mRNA-based cancer vaccine developed by BioNTech, encoding up to 20 patient-specific tumor neoantigens) is currently being tested in pancreatic cancer. In a phase I trial for resectable pancreatic ductal adenocarcinoma (PDAC), eight of 16 patients (“responders”) developed neoantigen-specific CD8⁺ T-cell responses and none recurred within 18 months post-treatment. Non-responders had a median recurrence-free survival (RFS) of 13.4 months [13].

Subsequent 3.2-year follow-up revealed that vaccine-induced CD8⁺ T-cell clones persisted long-term—with an average lifespan of 7.7 years, and ~20% of clones projected to survive decades. These cells exhibited tissue-resident memory and durable effector functions; vaccine-targeted clones were selectively lost in recurrent tumors [14]. This constitutes the longest follow-up demonstrating vaccine-induced T-cell clonotypes that may surpass host survival, addressing a critical hurdle in therapeutic cancer vaccination [14].

### Artificial intelligence (AI)-assisted neoepitope prediction

High-fidelity antigen prediction is essential. Integrating machine learning (e.g., NEO networks) with multi-omics datasets has improved neoepitope selection accuracy, reducing false positives/negatives and accelerating vaccine design timelines [13, 14]. Bioinformatics pipelines emphasize the need for prediction accuracy and manufacturing streamlining [15–17].

### Other promising paradigms

In KEYNOTE-942, the combination of personalized mRNA vaccine mRNA-4157 and pembrolizumab in resected high-risk melanoma reduced recurrence risk by ~50% and significantly extended RFS to ~75% over 2.5 years vs. 56% with pembrolizumab alone [18].

Personalized neoantigen mRNA vaccines in human papillomavirus (HPV)-positive head and neck squamous cell carcinoma also have a strong rational, eliciting antitumor activity in mice bearing HPV-expressing mEER oropharyngeal and TC-1 lung carcinomas, evidenced by increases in CD8^+^ T cells and T-cell clonality in the tumor microenvironment (TME) [19].

A phase I peptide neoantigen vaccine trial in resected high-risk renal cell carcinoma (RCC) demonstrated that vaccinated patients had antigen responses in 78%, and none recurred over a median 40 months follow-up [20]. In the advanced setting, success from IMA901 vaccine containing multiple tumor-associated peptides (TUMAPs) that are naturally present in human cancers was more limited, with one out of ten patients in this phase I/II study experiencing a partial response and 8 in 10 with stable disease. Interestingly, half patients showed a vaccine-induced T-cell response against at least one human leukocyte antigen (HLA) class I-restricted TUMAP and two patients had T-cell responses to multiple TUMAPs [21].

## Challenges and opportunities

### Tumor heterogeneity and immune evasion

Despite the promising immunogenicity of cancer vaccines, multiple tumour-intrinsic and extrinsic immunosuppressive mechanisms can blunt the efficacy of vaccine-induced T-cell responses. One major barrier is infiltration of regulatory T cells (Tregs) into the TME. Tregs, which express high levels of CD25, CTLA-4, and FOXP3, can suppress effector T-cell function through interleukin-2 (IL-2) consumption, inhibitory cytokines (e.g., IL-10, TGF-β), and modulation of DCs [22, 23]. Their accumulation is often enhanced following vaccination, particularly in the absence of checkpoint blockade or adjuvant strategies designed to modulate Treg activity [24].

Another critical mechanism is upregulation of immune checkpoint ligands, particularly PD-L1 (programmed death-ligand 1), on tumor cells or APCs in response to interferon-γ produced by vaccine-activated T cells. This feedback loop leads to T-cell exhaustion, marked by reduced cytokine production, proliferation, and cytolytic activity. Preclinical and clinical studies have shown that PD-L1 expression increases following vaccination and limits the durability of antitumor responses unless PD-1/PD-L1 blockade is co-administered [25, 26].

Additionally, tumors evolve via immunoediting and may lose target antigens or downregulate antigen presentation machinery. In this process, immune pressure from neoantigen-specific T cells leads to selective outgrowth of tumor cell clones that no longer express the targeted antigen, either due to mutations, HLA downregulation, or epigenetic silencing [27, 28]. This phenomenon has been observed in both mRNA and peptide vaccine trials, where recurrent tumors lose vaccine-targeted neoepitopes, allowing immune escape despite initial response [29].

Further suppression can arise from metabolic competition in the TME—e.g., hypoxia, high lactate, or tryptophan depletion via indoleamine 2,3-dioxygenase (IDO)—which impairs T-cell survival and effector function [30]. Collectively, these suppressive networks necessitate combination strategies that pair vaccines with checkpoint inhibitors, Treg depletion, metabolic modulators, or multi-epitope vaccine design to counteract escape and achieve sustained tumor control [31].

### Manufacturing and scale

Despite decreasing sequencing costs, personalized vaccine manufacturing remains complex, time-intensive, and expensive. Strategies to centralize production and automate pipelines—as seen with OncoVAX’s cGMP pipeline—can enhance scalability [23]. There’s a need for streamlined regulatory pathways to support individualized product development.

### Biomarkers and response metrics

Effective immune correlates [e.g., ELISpot, T cell receptor (TCR) clonotyping] and clinical endpoints are crucial to select responsive patients, adjust dosing, and assess efficacy [32, 33]. Integration of immunological and clinical biomarkers is key for appropriate patient selection to maximize clinical benefit.

### Regulatory and ethical considerations

Individualized vaccines challenge conventional regulatory frameworks. Novel adaptive clinical trial designs, expedited pathways, and real-world data integration are vital to facilitate timely approval. Several lessons can be learned from the existing framework for viral design and endpoints in licensing therapeutic HPV16/18 vaccines to prevent cervical cancer [34].

### Combination immunotherapy approaches

**Figure 1:**
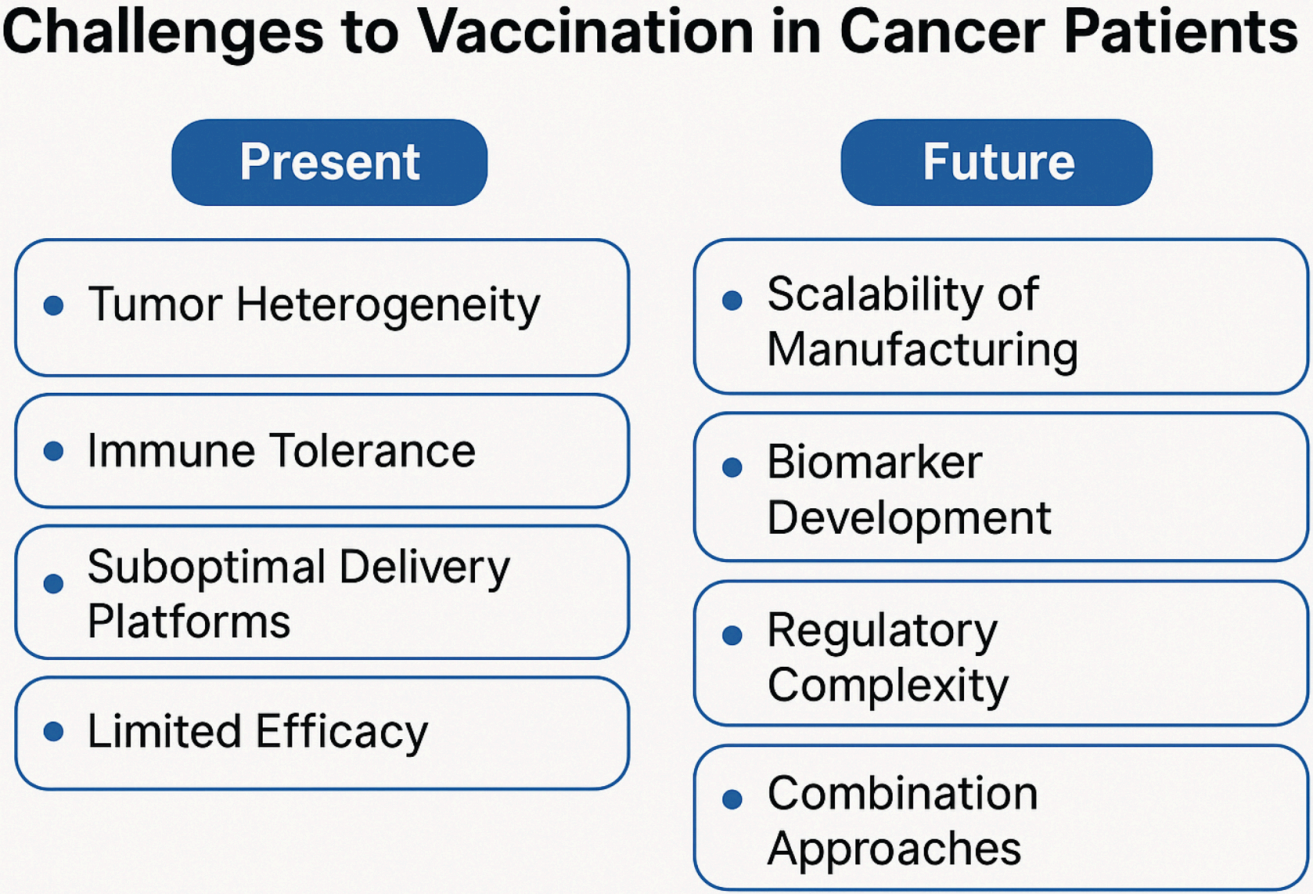
Key present and future challenges to vaccination in cancer patients.

Vaccines may be most effective when combined with checkpoint blockade, targeted adjuvants (e.g., TLR agonists), or immunomodulators. Enhanced synergy has been observed in several tumor types, including melanoma (vaccine + pembrolizumab), prostate cancer (PROSTVAC + nivolumab), cervical cancer (PDS0101 + PDS01ADC + PD-L1/TGF-β inhibition, MEDI0457 + durvalumab, ISA101 + nivolumab), head and neck cancer (anti-MAGED4B/FJX1 + anti-PD-1) and supports further development of engineered combination strategies [18, 35–39]. Key present and future challenges to vaccination in cancer patients are illustrated in Figure 1.

## Toward a translational ecosystem

Achieving meaningful and lasting clinical impact from cancer vaccines will require the establishment of a fully integrated translational ecosystem, in which each step of the vaccine development and delivery pipeline is optimized and seamlessly connected. This includes widespread access to rapid, high-quality tumor sequencing and standardized bioinformatics workflows capable of accurately predicting immunogenic neoantigens in clinically actionable timeframes. Good Manufacturing Practice (GMP)-compliant manufacturing facilities must be scalable and distributed to ensure timely production and equitable access, while robust clinical trial networks are needed to evaluate new vaccine candidates efficiently across diverse populations. Regulatory frameworks must adapt to the unique demands of individualized therapies, allowing for streamlined approvals while maintaining rigorous safety and quality standards. National initiatives, such as coordinated cancer vaccine programs and dedicated translational hubs, can act as accelerators by pooling resources, harmonizing protocols, and fostering data-sharing across institutions. Equally crucial is sustained collaboration among academia, biotech and pharmaceutical companies, government agencies, and global health organizations. Such cross-sector partnerships will be essential not only for scaling production and distribution but also for ensuring that personalized cancer vaccines are accessible to patients worldwide, regardless of geographic or economic constraints [40].

## Future perspectives

The trajectory of cancer vaccine development is poised to shift from individualized feasibility studies to scalable, clinically integrated therapies. Multiple converging advances—in immunogenomics, AI, synthetic biology, and regulatory policy—are reshaping both the scientific and infrastructural landscape of cancer immunotherapy.

### Personalized neoantigen vaccines will become routine in early-stage disease

While most personalized cancer vaccines have historically been trialed in advanced or refractory tumors—settings where the tumor burden is high and immune suppression is often profound—emerging evidence suggests their most effective role may lie in the adjuvant or neoadjuvant setting. In these contexts, the presence of minimal residual disease provides a substantially lower immunological barrier to eradication, enabling vaccine-induced T cells to efficiently seek out and destroy disseminated tumor cells before they establish macroscopic recurrence. Moreover, early-stage administration allows for integration with curative-intent surgery, chemotherapy, or radiotherapy, leveraging the immune-stimulating effects of cytoreductive therapies. Early vaccine intervention can amplify long-lived T-cell memory, delay or prevent relapse, and potentially reduce dependence on long-term systemic therapies such as checkpoint inhibitors. Over time, as predictive biomarkers improve and manufacturing pipelines become faster and more cost-effective, early-stage personalized vaccination could transition from a niche experimental strategy to a standard component of multimodal oncologic care.

### From personalized to semi-personalized: shared neoepitopes, hotspot mutations, and gene fusions

An emerging hybrid approach to cancer vaccination seeks to bridge the gap between fully individualized neoantigen vaccines and standardized “off-the-shelf” platforms by targeting shared or recurrent neoepitopes—peptide sequences that arise from common driver mutations and are presented in the context of frequently occurring HLA alleles [41]. The practical distinctions between fully personalized, semi-personalized, and “off-the-shelf” cancer vaccine approaches—particularly in terms of manufacturing and clinical feasibility—are summarized in Table 2. Such “public” neoantigens, including those derived from hotspot mutations in genes like *KRAS*, *TP53*, *BRAF*, and *PIK3CA*, are present across multiple patients and tumor types, enabling the design of semi-personalized vaccines that preserve much of the tumor specificity and immunogenicity of bespoke vaccines while dramatically improving manufacturing scalability and cost-efficiency. By incorporating panels of these recurrent targets into modular vaccine backbones, it becomes possible to match patients to pre-manufactured formulations based on their tumor mutation profile and HLA type, reducing the turnaround time from sequencing to treatment from months to days or weeks.

**Table 2:** Comparative overview of cancer vaccine modalities.

**Feature**	**Fully personalized**	**Semi-personalized**	**Off-the-shelf**
Antigen source	Unique neoantigens from each patient’s tumor	Shared neoepitopes (e.g., hotspot mutations, public neoantigens)	Tumor-associated antigens (TAAs) are common across patients
HLA matching	Patient-specific HLA binding predictions	Designed for frequent HLA alleles (e.g., HLA-A*02:01)	Broad population, minimal HLA stratification
Manufacturing lead time	6–12 weeks (custom synthesis, QC, formulation)	1–3 weeks (batch-based pre-manufactured panels)	Immediate (pre-made and stocked)
Production scale	Individual (*n* = 1)	Small-to-medium batches matched to subgroups	Mass production possible
Cost per dose (estimated)	$$$$ (high; $100k+ per patient)	$$–$$$ (moderate; depends on HLA/mutation match rates)	$ (low; standardized production)
Regulatory pathway	Complex; often under IND or adaptive frameworks	More streamlined; batch-based approval possible	Conventional biologics license application (BLA)
Example technologies	mRNA vaccines tailored to private neoantigens	mRNA or peptide vaccines targeting KRAS G12D, TP53 R175H, etc.	PROSTVAC, NY-ESO-1, HPV E6/E7 vaccines
Clinical flexibility	Maximal specificity; can target unique mutanomes	Balances specificity and scalability	Low specificity; limited by immune tolerance
Turnaround from biopsy to dosing	~8–12 weeks	~2–3 weeks	0 weeks
Challenges	High cost, time, and individualized QA/QC	HLA restrictions, partial personalization	Poor immunogenicity, immune tolerance

HLA: human leukocyte antigen; HPV: human papillomavirus; mRNA: messenger ribonucleic acid.

Besides point mutations, gene fusions can be exploited to create neoantigen-based vaccines, as in the case of sarcomas, particularly Ewing family tumors, harboring EWS-FLI1, EWS-ERG, EWS-WT1 fusions, and alveolar rhabdomyosarcoma with PAX7-FOXO1 fusions. Indeed, in a single-center analysis of 182 tumors with EWSR1 fusions, three‑quarters of Ewing sarcoma and DSRCT tumors shared a common breakpoint motif (EWSR1 exon 7 sequence “SQQSSSYGQQ–”) fused to defined partner sequences from genes such as *FLI1*, *ERG*, or *WT1* [42]. These conserved fusion‑junction peptides may serve as neoantigen candidates for personalized immunotherapy (for example, mRNA vaccines or immune monitoring), replacing or complementing conventional diagnostics. The availability of these peptide sequences, together with HLA‑binding prediction, provides a blueprint for targeting fusion‑specific epitopes in sarcoma. While the work was essentially descriptive and focused on establishing a workflow rather than demonstrating therapeutic efficacy, it provides valuable insight into the “neoantigen space” of EWSR1‑fusion tumors and supports the notion that routine clinical fusion detection can feed immunotherapy pipelines [42]. In a single‑arm pilot study, 52 patients with translocation positive, recurrent, or metastatic Ewing’s sarcoma family of tumors or alveolar rhabdomyosarcoma underwent leukapheresis pre‑chemotherapy; after standard multimodal cytoreduction, 30 of them initiated immunotherapy comprising autologous T cells, influenza vaccination, and DCs pulsed with tumor‑specific translocation peptides (and HLA‑A2‑restricted peptide E7), with cohorts receiving varying doses of recombinant human IL-22 [43]. The therapy was well tolerated, flu responses were robust (in all patients), but only ~39% mounted responses to the tumor fusion peptide and ~25% of HLA‑A2^+^ patients to E7. In an intention‑to‑treat analysis (median follow‑up 7.3 years), the 5‑year overall survival was 31% for all apheresed patients and improved to 43% among those who received immunotherapy. Thus, integrating immunotherapy post‑chemotherapy remission is scientifically feasible and clinically practicable; however, efficacy remains modest, with the need to improve antigen selection and DC potency. While limited by non-randomized designs, small sample sizes, heterogeneous disease types and treatments, and the relatively low tumor‑peptide immunogenicity observed, these studies strengthen the translational bridge between molecular diagnostics and immunotherapeutic design in rare sarcomas, and set the stage for more potent antigen‑ and immune‑engineered approaches in the near future.

Collectively, preclinical and early-phase clinical studies have shown that shared neoepitope vaccines can elicit potent T-cell responses capable of recognizing and killing tumor cells in diverse patient populations. As bioinformatic prediction models continue to refine epitope selection and coverage algorithms, this strategy may complement purely personalized approaches, offering a practical pathway to broaden access while retaining the precision of antigen targeting.

### AI-powered vaccine design and adaptive clinical trials

The integration of AI and machine learning into cancer vaccine development is rapidly redefining antigen discovery and optimization. Advanced deep learning architectures—trained on massive datasets of peptide-MHC binding affinities, T-cell receptor recognition patterns, and tumor immunopeptidomes—can now predict not only the likelihood that a given peptide will be presented on tumor cells, but also its immunogenic potential, stability in the MHC groove, and susceptibility to immune escape through tumor evolution. These models are increasingly incorporating structural modeling, such as AlphaFold-derived protein conformations, to refine predictions of antigen presentation and cross-reactivity. Furthermore, AI algorithms can assess HLA allele frequencies across global populations to design antigen panels that maximize coverage and minimize exclusion of underrepresented patient groups. When these predictive platforms are paired with adaptive clinical trial designs—frameworks that allow real-time modification of trial arms, dosing regimens, or antigen composition based on interim immunogenicity and efficacy data—cancer vaccine development can shift from a static, linear process to a dynamic, feedback-driven pipeline. This could enable near-real-time personalization of vaccine content within broader population-level trials, accelerating translation, reducing costs, and ultimately making bespoke immunotherapies accessible to far larger patient cohorts.

### Integrated vaccine-immunotherapy combinations

Future oncologic treatment paradigms are expected to position therapeutic cancer vaccines as central elements within multi-agent immunotherapy regimens. Rather than serving as standalone interventions, vaccines will likely be deployed to prime and expand tumor-specific T-cell populations, which can then be further amplified and sustained through synergistic agents. Checkpoint inhibitors, such as anti-PD-1/PD-L1 and anti-CTLA-4 antibodies, can release the brakes on these vaccine-induced T cells, enhancing their persistence and cytotoxic function. Pattern-recognition receptor agonists, including stimulator of interferon genes (STING) and TLR agonists, can act as potent immune adjuvants by promoting DC activation and pro-inflammatory cytokine production, thereby improving antigen presentation and immune priming. Bispecific antibodies, capable of redirecting T cells to tumor cells regardless of native TCR specificity, offer another means of augmenting vaccine-driven immunity, especially against heterogeneous tumor populations. Engineered T-cell therapies, such as TCR-transduced or CAR-modified lymphocytes, could be combined with vaccines to generate a dual wave of immune pressure—engineered cells providing immediate cytotoxicity while vaccines sustain endogenous immunity and limit antigen escape. In this integrated framework, the vaccine functions as both an initiator and a maintainer of anti-tumor immunity, transforming immunotherapy into a coordinated, multi-pronged assault that addresses the temporal and spatial complexity of tumor evolution.

### Decentralized manufacturing and rapid-response pipelines

The unprecedented global scale-up of mRNA vaccine production during the COVID-19 pandemic demonstrated that complex biologics can be manufactured, quality-controlled, and distributed at speed when infrastructure, logistics, and regulatory pathways are aligned. Translating this model to cancer immunotherapy could transform personalized vaccine accessibility. By establishing regional GMP-certified manufacturing hubs equipped with modular, automated synthesis and formulation platforms, patient-specific vaccines could be produced and released within a matter of weeks—rather than the months often required in current centralized pipelines. Such decentralization would reduce geographic inequities, allow closer integration of vaccine production with local clinical sequencing facilities, and enable rapid iteration of vaccine designs in response to evolving tumor antigen profiles or emergent resistance mutations. Advances in closed-system manufacturing, real-time quality analytics, and digital chain-of-custody systems would further ensure batch consistency, safety, and traceability across distributed sites. In the long term, coupling these hubs to national or international neoantigen databases and AI-driven design tools could support a “just-in-time” immunotherapy ecosystem, where highly individualized cancer vaccines are designed, manufactured, and administered with unprecedented speed and scale.

### Preventive cancer vaccines and immunoprevention

Looking further ahead, the field is beginning to explore the concept of immunoprevention—vaccination strategies aimed not at treating established tumors, but at intercepting cancer development in individuals at elevated risk. This paradigm shift would build on the successes of existing prophylactic vaccines, such as HPV and HBV vaccines, which have already demonstrated population-level reductions in virus-associated malignancies. Preventive cancer vaccines could target individuals with hereditary cancer syndromes (e.g., Lynch syndrome, BRCA1/2 mutations) or those with chronic inflammatory states and premalignant lesions, where tumorigenesis follows a predictable trajectory. Advances in genomics and longitudinal liquid biopsy monitoring may soon allow for the early identification of emerging driver mutations or clonal expansions in these populations, enabling the design of vaccines that preemptively target these antigens before malignant transformation occurs. In parallel, immunopreventive approaches could be applied in high-incidence regions, using panels of shared neoantigens or hotspot mutations tailored to prevalent cancer types and local HLA profiles. While challenges remain—including ensuring long-term safety, balancing immune activation with tolerance, and conducting lengthy prospective trials—the potential impact of shifting cancer vaccination from a reactive to a preventive measure could be transformative, reducing incidence, mortality, and healthcare costs on a global scale.

### Safety signals and mitigation strategies from COVID-19 and cancer vaccine trials

Both COVID-19 vaccines and cancer vaccines have demonstrated generally favorable safety profiles; however, notable safety signals have emerged that warrant careful monitoring and proactive mitigation. Reactogenicity, including transient injection site pain, fatigue, fever, and myalgias, is the most common adverse event associated with mRNA-based vaccines (e.g., BNT162b2, mRNA-1273) and is typically self-limiting and indicative of immune activation [44, 45]. More concerning are rare but serious immune-related adverse events. For COVID-19 mRNA vaccines, cases of myocarditis, especially in young males, have been reported post-vaccination [46]. Additionally, theoretical concerns about type I interferon (IFN-I)-mediated autoimmunity have been raised, given that excessive IFN-I signaling is implicated in autoimmune diseases such as lupus and type 1 diabetes [47, 48].

In therapeutic cancer vaccine trials, safety issues may arise from exaggerated immune activation, including cytokine release syndrome (CRS) or immune-mediated tissue damage, particularly when vaccines are combined with checkpoint inhibitors or strong adjuvants [6, 26]. DC vaccines, peptide vaccines, and mRNA platforms have shown acceptable safety profiles in early-phase trials, but ongoing vigilance is required. Mitigation strategies include: a) use of nucleoside-modified mRNA (e.g., pseudouridine) to suppress excessive innate immune stimulation [49]; b) optimization of LNP formulations to modulate delivery and minimize systemic exposure; c) dose-escalation and step-up dosing protocols to prevent abrupt cytokine surges; d) immune monitoring through cytokine panels, autoantibody testing, and T-cell profiling in early-phase trials; e) exclusion of patients with active autoimmune disease in initial cohorts to minimize risk; and f) careful combination therapy design, avoiding synergistic toxicity when pairing with checkpoint blockade or immunostimulatory agents [50].

As both preventive and therapeutic vaccines become increasingly personalized and potent, continued post-marketing surveillance, adaptive trial designs, and long-term follow-up will be essential to ensure safety across diverse populations.

### Global access and equity considerations

Cancer vaccines should not remain the privilege of patients in high-resource settings. Achieving true global equity will require a coordinated effort to address disparities in infrastructure, affordability, and regulatory readiness [51]. Regional manufacturing capacity—particularly in low- and middle-income countries (LMICs)—is essential to minimize dependency on centralized facilities in wealthier nations, which can lead to prohibitive costs and supply bottlenecks. This can be facilitated through technology transfer agreements, workforce training programs, and investment in modular GMP-certified production units adaptable to local needs. Equally important is the simplification of tumor sequencing and bioinformatics pipelines, enabling smaller healthcare systems to efficiently identify relevant neoantigens without requiring costly, specialized infrastructure. Regulatory harmonization across countries, supported by global health organizations, would reduce duplicative approval processes, accelerate vaccine rollout, and ensure adherence to consistent quality and safety standards. Addressing affordability will also demand innovative financing models, such as tiered pricing.

## Conclusions

Therapeutic cancer vaccines have transformed from speculative therapies to tangible clinical contenders. Personalized neoantigen vaccines, empowered by mRNA delivery technologies and guided by AI-based antigen selection, have generated long-lasting T-cell memory and produced encouraging clinical benefits across diverse cancer types. Addressing manufacturing, regulatory, immunological, and ethical challenges through cross-sectoral translational infrastructure will be key. With momentum from promising clinical trials and emerging ecosystems, cancer vaccines are poised to become central components in precision oncology.

## Abbreviations

AI: artificial intelligence
APCs: antigen-presenting cells
DC: dendritic cell
GM-CSF: granulocyte-macrophage colony-stimulating factor
GMP: Good Manufacturing Practice
HLA: human leukocyte antigen
HPV: human papillomavirus
HSP: heat shock protein
HSV-1: herpes simplex virus type 1
IFN-I: type I interferon
IL-2: interleukin-2
LNP: lipid nanoparticle
MHC: major histocompatibility complex
mRNA: messenger ribonucleic acid
PAP: prostate acid phosphatase
PDAC: pancreatic ductal adenocarcinoma
PD-L1: programmed death-ligand 1
RCC: renal cell carcinoma
RFS: recurrence-free survival
STING: stimulator of interferon genes
TAAs: tumor-associated antigens
TCR: T cell receptor
TLR: toll-like receptor
TME: tumor microenvironment
Tregs: regulatory T cells
TUMAPs: tumor-associated peptides
T-VEC: talimogene laherparepvec

## Declarations

### Copyright

© The Author(s) 2025.
